# Circulating Tumour DNA in Melanoma—Clinic Ready?

**DOI:** 10.1007/s11912-021-01151-6

**Published:** 2022-02-08

**Authors:** Ann Tivey, Fiona Britton, Julie-Ann Scott, Dominic Rothwell, Paul Lorigan, Rebecca Lee

**Affiliations:** 1 The Christie NHS Foundation Trust, Wilmslow Road, Manchester, M20 4BX UK; 2 Division of Cancer Sciences, The University of Manchester, Oxford Road, Manchester, M13 9PL UK; 3 Nucleic Acids Biomarker Team, Cancer Research UK Manchester Institute, Cancer Biomarker Centre, The University of Manchester, Alderley Park, SK10 4TG UK; 4 Department of Medical Oncology, The Christie NHS Foundation Trust, Wilmslow Road, Manchester, M20 4BX UK

**Keywords:** ctDNA, Liquid biopsies, Melanoma, Immunotherapy, Targeted therapy, Biomarkers

## Abstract

**Purpose of Review:**

Liquid biopsies, including circulating tumour DNA (ctDNA), can inform a variety of clinical questions. This review examines the potential role of ctDNA as a clinical tool to inform clinical decision-making from early to late stage cutaneous melanoma.

**Recent Findings:**

In pre-clinical studies, ctDNA has been shown to detect minimal residual disease and molecular relapse; predict and monitor response to therapy; and identify key resistance mechanisms. Here, we examine the potential utility of ctDNA and discuss its limitations for use in patients with melanoma. We present novel clinical trials, which are testing its value as a tool to augment clinical decision-making. Finally, we discuss the steps that are needed to ensure that ctDNA is used optimally in order to improve outcomes for patients with melanoma.

**Summary:**

Preclinical studies have shown that ctDNA has huge potential to provide real-time information about disease status in patients with melanoma. It is now time to test it rigorously within clinical trials to assess how it can be optimally used to benefit patients in the clinic.

## Introduction

There have been huge therapeutic advances in the treatment of both early and advanced melanoma over the past two decades, with targeted therapies and immune checkpoint inhibitors (ICI) significantly improving progression-free and overall survival. In parallel, with the development of effective treatment options, the concept of ‘precision’ or ‘personalised’ medicine has gained traction, whereby timing and choice of treatment are tailored to an individual patient and their tumour. Blood-based biomarkers such as circulating tumour DNA (ctDNA), circulating tumour cells (CTCs) and other ‘liquid biopsies’ have the potential to play a key role in enabling precision strategies to be delivered. This review will focus on the use of ctDNA for clinical applications of liquid biopsies; however, assays including CTC enumeration and analysis can provide additional information about the cancer such as gene expression/protein changes, which will also be important for the clinic.

Cell-free DNA (cfDNA) is DNA that freely circulates in the bloodstream and consists of fragments that are on average 140 to 170 base pairs (bp) long [1]. CfDNA is found in the blood stream of healthy subjects at an average concentration of 2 ng/ml (range 1–6 ng/ml) and in patients with lung cancer an average of 8 ng/ml (range 1–41 ng/ml) [2]. The mechanisms of cfDNA release are not completely understood; however, it is thought it is produced by cell necrosis, apoptosis and secretion from macrophages that have phagocytosed cells [1, 3]. Circulating tumour DNA originates from cancer cells and contributes to total cfDNA in the blood, thereby increasing the concentration of cfDNA when cancer is present. Studies have shown that there is enrichment of cfDNA fragment sizes of between 90 and 150 base pairs in patients with cancer compared to healthy volunteer blood but also a lower enrichment at 250–300 base pairs [4].

Mandel and Métais first identified circulating nucleic acids in the blood stream in 1948; however, it was not until 1994 that their potential utility as biomarkers for cancer detection and monitoring was realised [1]. At that time, presence of mutated *KRAS* ctDNA sequences were identified in blood of patients with pancreatic cancer with *KRAS* mutant tumours [5••]. Many studies since then have examined the role of ctDNA as a ‘liquid biopsy’ which can predict response to treatment, monitor clinical response, identify timing of disease progression and elucidate mechanisms of resistance to therapy.

**Fig. 1:**
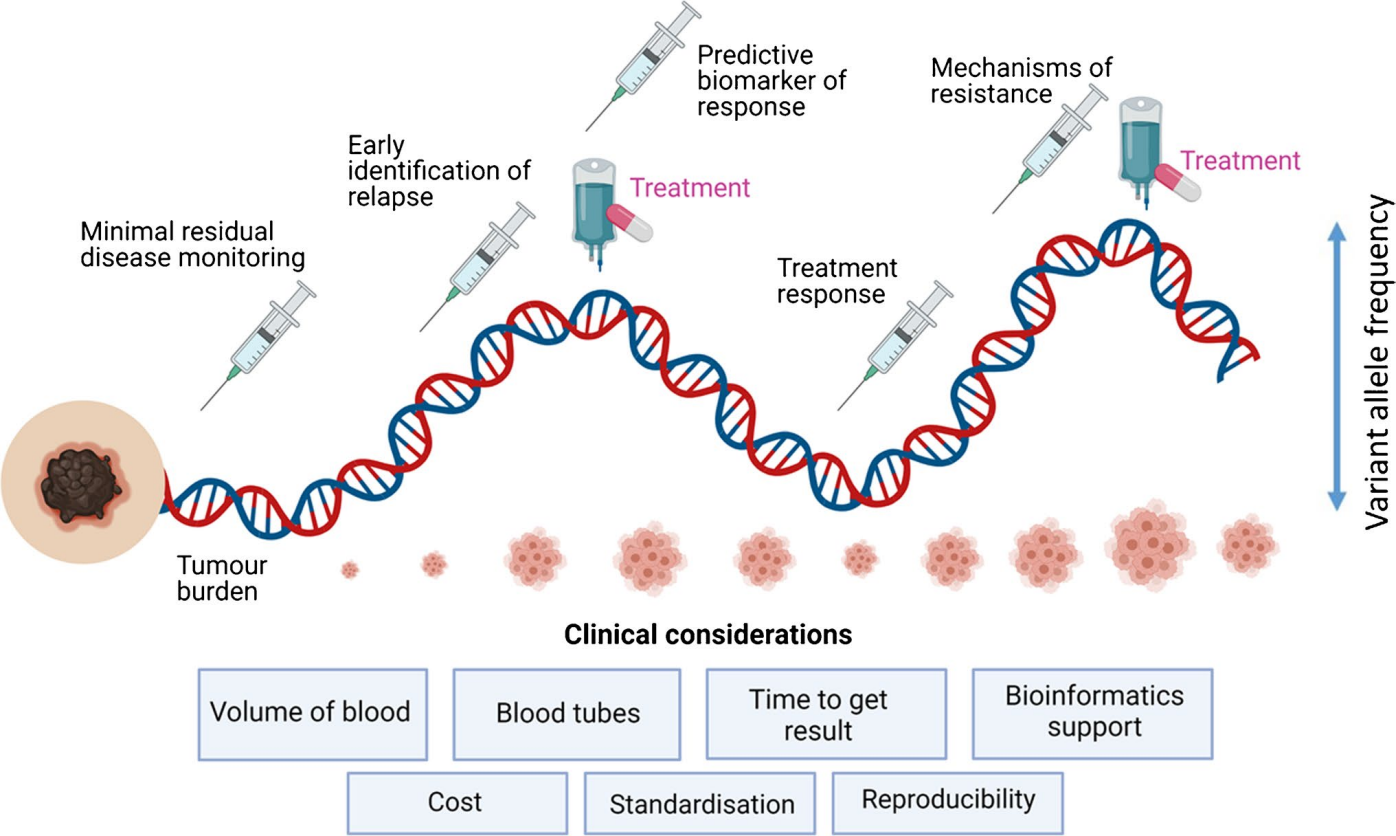
Potential clinical utility of ctDNA throughout melanoma disease stages

In this review, we will discuss the potential role of ctDNA as a clinical tool to inform clinical decision-making from early to late stage cutaneous melanoma. We reflect on the current evidence base for use of ctDNA in the clinic and its limitations. Finally, we will examine trials that are using ctDNA to inform real-time clinical decisions and propose how ctDNA could be developed to aid future treatment strategies (Fig. 1).

## Analysis of Circulating Tumour DNA

There are a huge number of different methods to analyse ctDNA, from digital techniques analysing specific point mutations, to targeted panels (generally analysing up to 1000 genes), to broader approaches using whole exome sequencing (WES) and methylation-based assays. These have been extensively reviewed elsewhere and therefore will not be discussed in detail in this review [1, 6, 7]. However, it is important to note that determining the right assay to use for different clinical scenarios is critical when considering the application of ctDNA to the clinic. For example, in developing an assay for minimal residual disease detection (MRD), broad but low depth whole exome sequencing is unlikely to have sufficient sensitivity to identify micro-metastatic disease; however, this technique may be more useful in characterising novel resistance mechanisms to therapy. Each assay has advantages and limitations, which affect its utility for a specific scenario. Newer techniques such as MRD-EDGE using machine learning-based denoising and an expanded feature space including fragmentomics and allelic frequency of germline single nucleotide polymorphisms are however changing this paradigm. Thus, when designing trials using ctDNA, clinicians should ask themselves what information is most important to obtain for the patient at that particular stage of disease in order to address the clinical question, then tailor the ctDNA assay accordingly. It will also be important, if ctDNA moves beyond the trial setting and into the clinic, that clinicians understand the performance of the test they are using in their patient population and appreciate its limitations if they are to make treatment decisions on the basis of its results. In addition, in some cancers, driver mutations (e.g. BRAF V600) are easier to identify than others where multi-regional sequencing might be required to identify truncal mutations. This impacts on the ability to select single-point mutations to assess tumour burden. Other clinical considerations include ease of sample collection and initial laboratory processing, requirement for bioinformatics support in interpreting the results and overall cost in performing the assays, especially if longitudinal monitoring is proposed.

## Identification of Minimal Residual Disease and Early Relapse Monitoring

Adjuvant therapy with either targeted (dabrafenib plus trametinib [D + T]) or ICI (pembrolizumab or nivolumab) is now standard of care for patients with stage III melanoma based on data showing improved relapse-free survival (RFS) [8–11]. However, these treatments are not without side effects and ICI, in particular, is associated with long-term endocrine toxicities, which may impact future quality of life for patients [12]. As such, it is important that adjuvant therapy is offered to patients who will gain the greatest benefit, and lower-risk patients spared from possible life-threatening and lifelong toxicities. There are now data on the prognostic role of ctDNA in patients with melanoma who have undergone curative-intent surgical resection at pre-operative, post-operative and post-adjuvant time points.

Detection of ctDNA pre-operatively in patients with stage III melanoma is predictive of relapse independent of standard American Joint Committee on Cancer (AJCC) staging [13•, 14••]. In a study of 58 patients with stage III (A–D) melanoma who had pre-operative plasma samples, 21 (36%) patients had detectable levels of ctDNA with 19 (90%) of these patients relapsing during the 20-month follow-up period (relapse-free survival (RFS) HR 2.9; 95% CI 1.5–5.6; P = 0.002). Of note, median time to relapse was 6.2 months for those with detectable ctDNA vs. 10.8 months in 18 (49%) of patients who relapsed within the follow-up period, but did not have detectable ctDNA pre-operatively [14••]. In parallel, another group examined whether pre-operative ctDNA levels in 119 patients with stage III (B–D) melanoma was associated with outcome following surgery. CtDNA was detected in pre-operative samples of 34% and 33% of patients in their discovery and validation cohorts, respectively. The time to distant metastatic relapse (DMFS) was shorter in patients with detectable ctDNA with a median of 6.2 months vs. 13.9 months (HR 1.59; 95% CI 1.0–2.52; *P* = 0.027) and 8.7 months vs. 14.5 months (HR 2.15; 95% CI 1.04–4.47, *P* = 0.014), in the discovery and validation cohorts, respectively [13•]. In addition, patients with detectable ctDNA had a median melanoma-specific survival (MSS) of 17.6 months compared with 49.4 months in those with undetectable levels (HR 2.11; 95% CI 1.20–3.71, *P* < 0.01) [13•]. This remained significant in multivariable analysis of characteristics including stage, number of lymph nodes, size of lymph nodes, and extranodal extension [13•]. All these studies used droplet digital PCR (ddPCR) to identify ctDNA in volumes of up to 5 ml of plasma. Based on these data, there is a potential role for ctDNA in augmenting current staging methods in order to predict survival of patients with stage III melanoma. Furthermore, results from ctDNA analyses expected to be reported from these pivotal trials may inform as to its role in predicting risk of recurrence, survival and response to adjuvant treatment.

Through serial assessments of ctDNA, it is possible to identify MRD and early disease relapse (molecular progression) following curative intent surgery for early stage melanoma. In a retrospective study of 161 patients with stage III melanoma and no radiological evidence of disease, detection of *BRAF/NRAS* mutant ctDNA (by ddPCR) at a single timepoint within 12 weeks of surgery was associated with poor outcome (disease-free Interval (DFI); HR 3.12; 95% CI 1.79–5.47; *P* < 0.0001, distant metastasis-free interval (DMFI); HR 3.22; 95% CI 1.80–5.79; *P* < 0.0001, OS HR 2.63; 95% CI 1.40–4.96; *P* = 0.003) [15••]. The presence of ctDNA remained a significant predictor of relapse and OS in multivariate analyses [15••]. These results were later validated in two prospective cohorts [14••]. Targeted amplicon-based next-generation sequencing (NGS) or pyrosequencing were used to identify mutations in formalin-fixed-paraffin-embedded (FFPE) tissue in order to develop bespoke ddPCR assays for specific point mutations. Detection of ctDNA within 12 weeks of surgery was a strong predictor of relapse (RFS HR 10; 95% CI 4.3–24; *P* < 0.001), with all but one patient across the two cohorts experiencing disease progression within the 20-month follow-up period [14••]. Critically, longitudinal monitoring improved the sensitivity of detecting relapse over time, with a number of patients who were negative at the first post-operative timepoint subsequently developing detectable ctDNA prior to relapse [14••]. There remains a challenge in the use of ctDNA to inform a decision to give adjuvant therapy immediately post-operatively as there may be false negative results with patients subsequently relapsing. Technical improvements in sensitivity of the assays may aid future decision-making at this timepoint, or longitudinal monitoring may offer an alternative strategy.

Evaluation of ctDNA in patients may also inform monitoring of response to adjuvant ICI following curative surgery. In a small study of 18 patients who received adjuvant ICI (anti-PD-1), 4/14 patients had detectable ctDNA post-operatively, and in 2 of these patients, ctDNA subsequently become undetectable during treatment with ICI [14••]. Critically, after 7 months of follow-up, there was no evidence of relapse, suggesting that ctDNA could be used to define early response to adjuvant treatment. Larger studies and longer-term follow-up are required to validate these results.

Based on retrospective and prospective data demonstrating that ctDNA could be used to longitudinally monitor for molecular recurrence in the post-operative setting, we developed the DETECTION phase II/III study (Circulating tumour DNA guidEd Therapy for stage IIB/C mElanoma after surgiCal resection; NCT04901988). The study aims to address a challenge in managing patients with stage II melanoma where the individual risk of death for patients with stage IIB/C melanoma is low, but because they are very common, they contribute to 30–50% of all melanoma deaths [16, 17]. CheckMate 76K (NCT04099251) and Keynote 716 (NCT03553836) are examining the role of adjuvant anti-PD-1 in preventing relapse of stage IIB/C melanoma. However, treating unselected patients upfront exposes a large number to toxicity and potential impact on quality of life, when their disease is already cured by surgery alone. In addition, there is a financial and resource cost to the health system. DETECTION uses ctDNA to monitor for MRD and molecular relapse following surgery for stage IIB/C melanoma. If ctDNA is detected, patients are randomised to standard of care, which is treatment upon clinical/radiological relapse or early treatment with nivolumab. It will prospectively assess whether ctDNA can identify disease recurrence earlier than standard of care and critically whether early treatment of micro-metastatic disease with ICI improves outcomes.

## Advanced Melanoma

CtDNA can also be used to guide treatment choice and monitor response in the setting of stage IV disease. A number of studies in advanced disease have shown that a decline in plasma ctDNA is associated with response and may often precede radiological evidence of progression [18, 19, 20•, 21, 22]. Levels of cfDNA have been shown to correlate with tumour burden on CT scan and levels of ctDNA have been shown to correspond with metabolic burden of disease as assessed by FDG-PET [20•, 23, 24]. However, there may be variation in ctDNA release according to disease site with patients with visceral, bone, or lymph node involvement exhibiting higher levels of ctDNA out of keeping with the metabolic disease burden as assessed by FDG-PET, whilst those with extensive subcutaneous disease or with brain metastases showed consistently low levels of ctDNA despite measurable disease [20•].

However, there is huge potential for ctDNA to be used as a tool to guide treatment decisions for patients receiving both targeted agents and ICI.

## Targeted Therapy

Targeted therapy with combination BRAF and MEK inhibitors is a key line of treatment in patients with advanced *BRAF* V600 mutant melanoma. Based on data from clinical trials showing improvement in both PFS and OS, these inhibitors are now approved as standard of care and are widely used in management of patients with stage IV melanoma [25–27]. A number of studies have shown that ctDNA can be used at baseline as a predictive biomarker of response to targeted therapy, an on-treatment biomarker of response/disease progression and a tool to identify mechanisms of resistance.

The baseline level of mutant *BRAF* in cfDNA has been shown to be a predictive biomarker of duration of therapy in patients treated with the BRAF/MEK inhibitors. In a pooled analysis from four clinical trials, enrolling patients with tissue confirmed *BRAF* V600E/K mutant melanoma; copies of mutant *BRAF* were identified in 76% (V600E) and 81% (V600K) of 732 baseline plasma samples using BEAMing (beads, emulsion, amplification). Its presence was associated with lower response rates compared to those patients where mutant *BRAF* was undetectable [19]. Furthermore, patients who had detectable mutant *BRAF* had a significantly shorter PFS and OS compared to those with undetectable levels [28]. This remained significant in multivariable analysis comparing baseline factors including lactase dehydrogenase (LDH) and performance status in 3 out of 4 of the studies [28]. More recently, a further validation study of patients treated in the COMBI-d and COMBI-MB trials of dabrafenib plus trametinib (COMBI-MB in patients with brain metastases) supported baseline detectable ctDNA as a predictor of PFS and OS on targeted therapy [29•]. Using a cut-off of 64 copies of ctDNA per millilitre (determined using ddPCR), they stratified patients as high and low risk. Patients with low- vs. high-risk disease had a significantly longer PFS 12.7 vs. 6.5 months (HR 1.74; 95% CI 1.37–2.21, *p* < 0.0001) and 35.1 vs. 13.4 months (HR 2.23; 95% CI 1.73–2.87, *p* < 0.0001) [29•].

There is also increasing evidence that ctDNA could be used to monitor patients on therapy, and that ctDNA changes may be detectable ahead of radiological or biochemical lactate dehydrogenase (LDH) change, giving an early indicator as to treatment efficacy or an early herald of disease progression. A study compared ctDNA vs. LDH in identifying disease progression within 15 days of confirmed radiological progression in 26 patients. In 82% of patients, ctDNA progression was detected vs. only 40% having an LDH rise, with a median difference in sensitivity of 42% (95% confidence interval, 27–58%; *p* < 0.001) [30]. A further study took longitudinal samples from 36 patients on targeted therapy, with 12 of these having detectable ctDNA levels prior to commencement of treatment [31]. There was a significant decrease in ctDNA in all of the patients (*p* < 0.01), with median time to becoming undetectable (*n* = 7) or < 1% (*n* = 5) of 13 days (range 6–40 days) [31]. An increase in the *BRAF* V600 mutant ctDNA fraction was detected prior to the clinical diagnosis of progressive disease in 12 out of 27 (44%) patients and simultaneously with PD in 7 out of 27 (26%) patients [31]. These data were subsequently confirmed in another study, which showed that ctDNA levels increased before radiological progression by a mean of 110 days [32].

Although one study did not support the use of ctDNA as an on-treatment biomarker of response to targeted therapy [31], larger studies have shown that it could be a useful tool [29•, 33]. One study investigated whether ctDNA levels of 10 copies per mL or higher in the first plasma sample since treatment initiation were predictive of PFS. They found that patients with undetectable ctDNA had a median PFS of 9 vs. 4 months in patients with detectable ctDNA (HR, 4.05; 95% CI, 1.56 to 10.53). A large analysis from the COMBI-d trial showed that 201/224 patients had detectable ctDNA at baseline, and 121/201 still had detectable ctDNA following 4 weeks of treatment. Disease control (complete response, partial response or stable disease) as best overall response was associated with 65/80 patients with conversion to undetectable levels vs. 63/118 patients with detectable ctDNA at week 4 (proportional-odds-likelihood-ratio test for association *P* = 0.0002) [29•]. In patients with high LDH only (above upper limit of normal), undetectable ctDNA at 4 weeks was significantly associated with PFS (HR 1.99; 95% CI 1.08–3.64, *p* = 0.027) and OS (HR 2.38; 95% CI 1.24–4.54, *p* = 0.0089) [29•]. Conversion to undetectable ctDNA at week 4 was independently associated with both PFS and OS in a Cox regression model that included clinical prognostics factors such as performance status and 3 or more organ sites with metastases [29•]. Taken together, these studies suggest that decreasing ctDNA levels on treatment are predictive of response to targeted therapy.

Based on the understanding that ctDNA could be used as a sensitive biomarker of response and progression on therapy, the DyNAMIc trial (circulating tumour DNA guided adaptive BRAF and MEK inhibitor therapy) has been developed. This is a pilot study testing feasibility of and examining whether adaptive therapy could improve PFS in patients on targeted therapy (E + B). Adaptive therapy relies on the competition between drug-sensitive and drug-resistant sub-clones to control overall tumour growth [34, 35]. It aims to stabilise tumour burden by allowing a significant population of treatment-sensitive cells to survive, which suppress proliferation of the less fit, resistant populations [34]. In vivo, this was shown to be more effective than intermittent scheduling [36], which was not shown to be beneficial in SWOG S1320 (median 9.0 months continuous vs. 5.5 months intermittent, *P* = 0.064, pre-specified two-sided *α* = 0.2) [37], although results from INTERIM (NCT03352947), which uses different timing of scheduling to SWOG S1320, are awaited. DyNAMIc will use the number of mutant *BRAF* copies/ml in cfDNA to determine when to stop and start drugs based on pre-specified thresholds which will be optimised in the trial.

Finally, a number of studies have shown that ctDNA can identify mechanisms of resistance to therapy. Studies have shown the emergence of *NRAS*, *MAP2K1*, *AKT1*, and *PIK3CA* mutations in patients treated with targeted therapy, which are known to be mechanisms of resistance to MAPK targeting therapy in melanoma and were associated with subsequent disease progression identified on CT scan [20•, 21, 38]. More recently, ARAF mutations, shown to be a novel mechanism of resistance to the selective RAF dimer (type II) inhibitor belvarafenib, have been described [39]. Therefore, ctDNA can be used to identify mechanisms of resistance, which could potentially aid patient selection or next-line therapy options.

## Immunotherapy

ICI have played a vital role in transforming clinical management and outlook of patients with melanoma. On the basis of data from randomised studies showing OS benefit, they are used as standard care treatment of metastatic melanoma and have more recently been shown to improve PFS in the adjuvant setting with OS data awaited [8, 10]. Both anti-PD-1 as single agents and combination anti-PD-1 plus anti-CTLA-4 inhibitors are currently used in the clinic. The combination of nivolumab plus ipilimumab (N + I) results in increased depth of response compared to single agents, however, is associated with increased toxicity. More recently, the RELATIVITY-047 Phase III trial showed improved PFS when relatlimab a LAG-3 antibody was combined with nivolumab vs. nivolumab alone [40]. Further ICI combinations are currently in early phase trials, and therefore in future, a key focus will be how to optimise sequencing of these therapies to extend PFS and OS. CtDNA may be a tool that could facilitate better decisions as to when to switch therapy.

Due to the heterogeneity of response to ICI, one of the main challenges is to identify predictive biomarkers, which could enable tailoring of treatment for the individual. A number of studies have shown that ctDNA can be used as a baseline biomarker of response to ICI [41, 42, 43••, 44, 45]. One study in 19 patients with *BRAF* and *NRAS* mutations showed that a baseline ctDNA level of < 10 copies/ml plasma prior to commencing ICI (anti-CTLA-4/anti-PD-1/combination) was associated with a significantly longer PFS (*P* = 0.009, relative risk = 5; 95% CI 1.8–13.8) [38]. In support of these findings, a further study in 141 patients treated with pembrolizumab found that patients with undetectable ctDNA at baseline vs. those with detectable levels of ctDNA using real-time PCR (*BRAF*) or ddPCR (*NRAS*) in 1 ml of plasma had a median PFS of 26 vs. 9 weeks (HR 0.47; *P* = 0.01) and OS not reached vs. 21.3 weeks (HR = 0.37; *P* = 0.005) [41]. This remained significant in multivariable analysis including LDH, CRP, number of metastatic sites (> 3), and performance status. Intriguingly, a recent study showed that baseline ctDNA appears to only be a biomarker of response in the first-line setting [44]. In two (albeit small) independent cohorts of patients with the majority receiving targeted therapy first line, baseline ctDNA of 20 copies/ml identified using ddPCR was predictive of longer PFS in first line but not second line in patients treated with ICI [44]. Whether this is the same if treated with ICI first line with new combinations such as anti-LAG3 plus anti-PD-1 will require further studies.

CtDNA has also been shown to be an on-treatment biomarker of response to ICI. Initially, rising ctDNA levels during longitudinal sampling of patients receiving ICI were shown to correlate with disease progression [42]. Subsequent studies have shown that clearance of ctDNA on treatment is a biomarker of PFS and OS [41, 43••, 45]. One study examined whether undetectable ctDNA levels (in ≥ 1 ml plasma using ddPCR) at baseline (group A, *n* = 36) and clearance of ctDNA from initially detectable baseline levels within 12 weeks of treatment with PD-1 antibodies (group B, *n* = 22) compared to those patients with persistently elevated ctDNA (group C, *n* = 18) were associated with response [45]. The median PFS was not reached in groups A and B and was 2.7 months for group C (*P* < 0.001, HR 0.09 for group A vs. C, and *P* < 0.001 HR 0.16 for group B vs. C). The median OS was not reached for groups A and B and was 9.2 months for group C (*P* < 0.001, HR 0.02 for group A vs. C and *P* < 0.001 0.14; for group B vs. C). This remained significant in multi-variable analysis of clinical features including LDH, performance status, tumour stage and disease volume. This is supported by another study in 85 patients (in 1 ml plasma using rtPCR and ddPCR), which showed that patients with undetectable ctDNA levels during follow-up (median 84 weeks) had improved survival compared to those with ctDNA present (adjusted HR for death 0.16; 95% CI 0.07–0.36, *P* < 0.001) [41]. A further study demonstrated that ctDNA dynamics as early as 2 weeks may be predictive of clinical response [43••]. No biological response defined as no reduction of ctDNA from baseline (alpha 2.5%) at 2 weeks was associated with a 0% response rate (0/10 patients) and a 0% PFS rate at 120 days (median PFS = 112 days) and a median OS of 130 days. In addition, all those who had a significant increase of ctDNA following an initial decline were found to have radiological progression at an average of 75 days in advance of radiologic detection of progression [43••].

CtDNA could also be a useful tool to clarify response in pseudo-progression, which is defined as an increase in the size of the primary tumour or the appearance of a new lesion followed by tumour regression, a phenomenon seen in approximately 10–30% of patients treated with ICI in melanoma [46, 47]. A favourable ctDNA profile (undetectable at baseline or conversion to undetectable by week 12) was able to differentiate true progression from pseudo-progression with a sensitivity of 90% (95% CI 68–99%) and specificity of 100% (95% CI 66–100%), which had better discrimination compared to LDH [48•]. This suggests that ctDNA is not simply a measure of tumour burden, but also reflects underlying tumour-immune response. The ability to distinguish pseudo-progression from true progression could reduce the need for repeat imaging and may prevent treatment being discontinued unnecessarily.

Moving towards using ctDNA as a potential tool to guide clinical decisions, the Circulating Tumour DNA Guided Switch (CAcTUS; NCT03808441) phase II feasibility trial in patients with *BRAF* mutant melanoma is examining whether response to ICI can be improved through pre-treatment with targeted therapy and switching treatment in response rather than resistance [49••]. Tumours responding to BRAF/MEK inhibitors have been shown to have increased T cell infiltration, improved T cell recognition of melanoma associated antigens and reduced production of immunosuppressive cytokines [50–53]. The SECOMBIT (NCT02631447) and EBIN (NCT03235245) studies are both assessing whether a pre-specified period of induction targeted therapy (12 weeks for EBIN and 8 weeks for SECOMBIT) results in improved survival [54, 55]. However, these strategies do not personalise treatment to the individual response of a patient and are at risk (particularly with 12 weeks of therapy) of patients already developing resistant clones which are more immune suppressive. CAcTUS personalises the decision to switch based on response seen in ctDNA. Patients on the intervention arm receive targeted therapy until there is evidence of response as defined by a decrease in mutant BRAF variant allele frequency (VAF) of ≥ 80% measured by ddPCR. The primary endpoint is logistical and assesses whether ctDNA results can be provided within 7 days with secondary endpoints providing a signal search as to efficacy of the strategy and insight into ctDNA monitoring.

## Central Nervous System Disease

Although ctDNA has been shown to be effective in identifying early disease progression, caution must be taken when assessing central nervous system (CNS) disease. A number of studies have reported undetectable ctDNA in patients with CNS-only metastases [23, 29•, 32, 56, 57, 58••]. In addition, the ability of plasma ctDNA to detect CNS progression (especially if extra-cranial disease is stable) has been shown to be variable, with one group reporting ctDNA progression associated with incidence of new brain metastases in 10/12 (83%) patients [18], whilst others have shown discordance in radiological and ctDNA progression [32]. Undetectable ctDNA on-therapy was associated with extracranial response (*P* < 0.01) but not intracranial response [58••]. This is an important consideration for on-treatment assessments where CNS-only progression may be missed, and also in the monitoring for early disease relapse.

CtDNA in CSF may provide an alternative tool for detection of CNS-only disease and has been shown to be more sensitive than plasma ctDNA in this context [59–61]. A number of case reports have suggested that CSF ctDNA may be useful for monitoring intracranial disease response or identifying targetable mutations, but there have not been any large studies performed in melanoma to validate these findings [62, 63]. In addition, CSF ctDNA may be useful in the diagnosis of leptomeningeal disease where radiological diagnosis can be challenging and cytological material in the CSF may be limited or even where present may be insufficient to permit analysis for *BRAF* mutations [64]. Due to the invasive nature of lumbar puncture, it is unlikely that ctDNA in CSF will be used for treatment monitoring; however, it may be useful to identify targetable mutations both at baseline and on progression and could be used as a predictor of future CNS disease.

## Conclusions

CtDNA is a powerful tool which has the potential to support tailored management of patients with melanoma throughout their treatment journey. By offering an accurate, easily obtainable method of assessing a patient’s disease status, ctDNA could enable precise and timely treatment decisions to be made which optimise efficacy and minimise unnecessary treatment burden. The potential for longitudinal sampling means that ctDNA can offer an early and dynamic assessment of treatment response. CtDNA can also characterise intra-tumour heterogeneity and differences in clonal response to therapy. Liquid biopsies are less invasive than tissue biopsies and therefore are useful if patients have disease in locations inaccessible/at high risk of complications. However, in general, ctDNA can only provide information on mutational or copy number changes, although new methods to infer gene expression based on nucleosome occupancy are being developed [65]. Other types of liquid biopsies such as CTCs used in parallel could give additional information regarding gene and protein expression.

In this review, we have highlighted a number of different potential applications for ctDNA. However, there are a number of challenges to overcome if ctDNA is to move into the clinic and be used to guide treatment decisions. There is anatomical variability in the extent of ctDNA release from particular sites including subcutaneous and intracranial disease, which may limit its utility in for certain patients [20•]. In addition, identifying MRD in particular can be technically challenging, although newer approaches using size selection to reduce background noise [4], improving bioinformatics pipelines [66], multiplexing [67] or analysing for methylation changes to increase the chances of identifying aberrations compared to normal cfDNA [68] could all improve detection. In addition, clonal haematopoiesis can also result in false positive results especially in genes associated with epigenetic modulators such as *DNMT3A* as well as *TP53*; therefore, some results may need to be interpreted with caution [69–71]*.*

Over the past 10 years, a huge number of techniques have been developed to analyse ctDNA. However, there is currently no standardised approach to compare them, which makes future-proofing clinical trials a challenge. Furthermore, though assays may be technically better at identifying ctDNA, their clinical benefit may be minimal. For example, assay A may improve detection of MRD in the laboratory, however may only detect disease recurrence 2 weeks before assay B in a patient, which is unlikely to result in clinical benefit. As a field, standards need to be developed and agreed upon to use for assay comparison. In addition, particularly in the context of MRD detection, there needs to be discussion and a framework developed as to how newer (e.g. more sensitive) assays could be substituted if proof-of principal for improved patient outcome was established with older assays.

Finally, although there have been many potential uses for ctDNA identified pre-clinically, it is critical that its clinical benefit is established using randomised-controlled trials. Being able to identify early progression or treatment resistance does not necessarily mean that treating early or switching early to a new treatment line will improve outcomes for the patient. Lessons can be learnt from the SWOG S0500 trial, which did not show OS improvement for patients with breast cancer with persistently increased CTCs after 21 days of first-line chemotherapy who were switched early to an alternative treatment and OV05/EORTC 5595, which did not show a survival benefit for early treatment of relapse on the basis of a raised CA125 [72, 73]. Thus the next stage of testing of ctDNA within clinical trials is crucial to ensure that ctDNA is optimally used to improve outcomes for patients with cancer.

CtDNA has huge potential to provide real-time information about tumour activity, disease status, treatment response/progression and mutational profile at any given time following melanoma diagnosis. It is now time to extensively test its value within the clinic in order to improve patient outcomes.
